# The Synthesis of Glycyrrhetinic Acid Derivatives Containing A Nitrogen Heterocycle and Their Antiproliferative Effects in Human Leukemia Cells

**DOI:** 10.3390/molecules15064439

**Published:** 2010-06-21

**Authors:** Yuan Gao, Xin Guo, Xiaojing Li, Dan Liu, Dandan Song, Ye Xu, Ming Sun, Yongkui Jing, Linxiang Zhao

**Affiliations:** 1 Key Laboratory of Structure-Based Drugs Design & Discovery of Ministry of Education, Shenyang Pharmaceutical University, Shenyang 110016, China; 2 Department of Pharmacology, Shenyang Pharmaceutical University, Shenyang 110016, China; 3 Mount Sinai School of Medicine, One Gustave L. Levy Place, New York, NY 10029, USA

**Keywords:** antiproliferative, glycyrrhetinic acid derivative, leukemia, nitrogen heterocycle

## Abstract

Fifteen novel glycyrrhetinic acid derivatives containing a nitrogen heterocycle at C-30 and with different A-ring substituents were designed and synthesized. All of these derivatives have improved antiproliferative effects against human HL-60 leukemia cells. Compounds with a cyano-enone functionality on the A-ring exhibit greater growth inhibitory effects, compared to those with a 2-hydroxymethylene-3-keto, an isoxazole, or a 2-cyano-3-keto group. *N*-(2-cyano-3,11-dioxoolean-1,12-dien-30-yl)-4-piperidyl piperidine (**9b**) was found to be two-fold more potent than methyl 2-cyano-3,11-dioxooleana-1,12- dien-30-oate (**CDODO-Me-11)**.

## 1. Introduction

18β-Glycyrrhetinic acid (**GA**, 3β-hydroxy-11-oxo-olean-12-ene-30-oic acid), the aglycone of glycyrrhizic acid isolated from licorice root, has been shown to have antitumor effects [1,2]. To improve its antitumor activities, some structural modifications were previously done. We found that introduction of an alkoxyimino group at position C-3 along with a free C-30 carboxyl group improved the antiproliferative effects of GA [3]. We and other groups have also found that introduction of a 2-cyano-1-en-3-one on the A-ring, modification of ring C by converting the 11-oxo-12-ene to 12-oxo-9(11)-en and/or methyl esterification of the C-30 carboxylic group, significantly improved the cytotoxic activities of GA [4,5,6]. Similar structural modifications have been performed in oleanolic acid and it was found that 2-cyano-3,12-dioxooleana-1,9(11)-dien-28-oic acid (CDDO) and its methyl ester CDDO-Me were potent cytotoxic agents against tumor cells [7,8,9]. 

To determine the importance of introducing a cyano-enone at the A-ring and methyl esterification at C30, glycyrrhetinic acid derivatives containing a nitrogen heterocycle at C-30 and with some additional modifications of the A-ring were designed and synthesized. The antiproliferative abilities of these compounds were tested in human HL-60 leukemia cells and compared with that of **GA**, methyl 2-cyano-3,11-dioxo- oleana-1,12-dien-30-oate (**CDODO-Me-11**) and methyl-2-cyano-3,12-dioxo- oleana- 1,9(11)-dien-30- oate (**CDODO-Me-12**), which have been found to be potent antileukemia agents [4]. The synthesis of these compounds and their abilities to inhibit growth of HL-60 cells are presented in this communication.

## 2. Results and Discussion

### 2.1. Chemistry

**Scheme 1:**
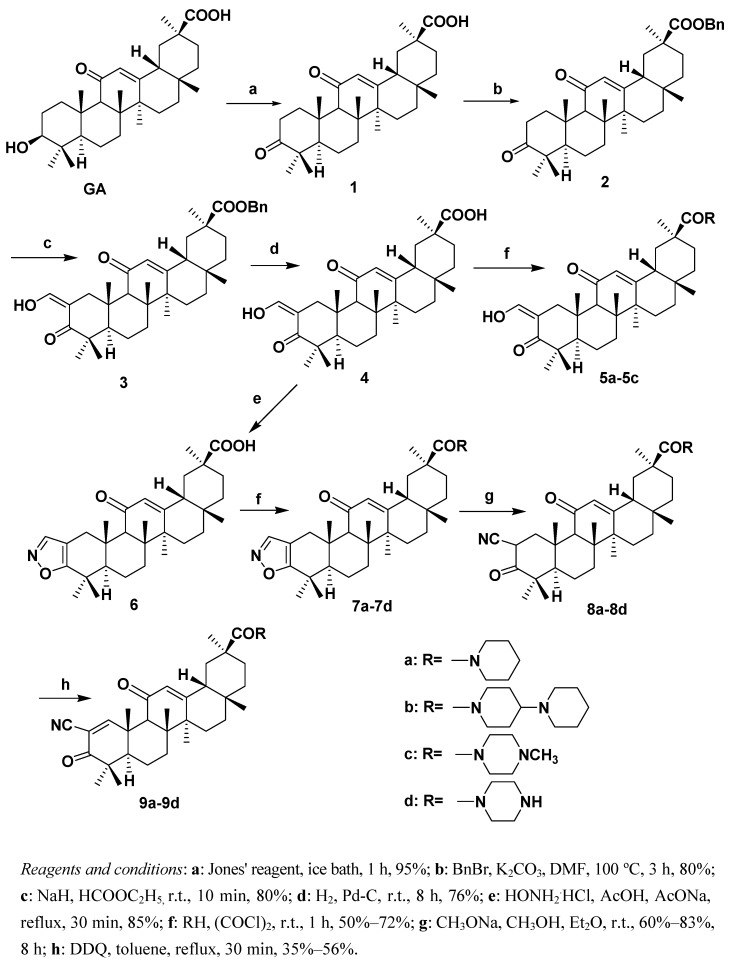
Synthetic routes to the target compounds.

Four groups of **GA** derivatives with a different A-ring structure and a replacement at the C-30 carboxylic group with a nitrogen heterocycle – piperidine, 4-piperidyl piperidine, 4-methyl piperazine, or piperazine – were synthesized starting from GA (Scheme 1**)**. Compounds **1-3** were obtained using a routine method [5,9,10]. A benzyl group instead of a methyl group was used to protect the C-30 carboxylic acid as halogenolysis of the methyl ester with LiI in DMF gave an 11-oxo-13(18)-en by-product in approximately 20% yield [8]. 

The benzyl group of compound **3** was removed under mild conditions to give the key intermediate **4 **in 76% yield, which was used as the precursor for generating the 2-hydroxymethylene-3-keto compounds **5a-5c** and the isoxazole **6**. Addition of oxalyl chloride to compound **4** in chloroform gave the corresponding acyl chloride**. **Amides **5a**-**5c** were prepared in 50%–72% yield by condensation between the acyl chloride and the corresponding nitrogen heterocycle. 

Using the same procedures employed for obtaining compounds **5a**-**5c**, the isoxazole amides **7a**-**7d** were obtained from isoxazole **6**, which was prepared in 85% yield by refluxing compound **4** with hydroxylamine hydrochloride in glacial acetic acid. Under basic conditions compounds **7a**-**7d** were isomerized to the corresponding 2-cyano-3-keto compounds **8a**-**8d**, that were then dehydrogenated using dichloro-5,6-dicyano-1,4-benzoquinone (DDQ) in toluene to give the cyanoenone amides **9a**-**9d **in fair to moderate yields. 

### 2.2. HL-60 cell growth inhibition activity

The abilities of compounds **5a-5c**, **7a-7d**, **8a-8d** and **9a-9d** to inhibit growth of HL-60 cells were determined and compared to that of **GA, CDODO-Me-11** and **CDODO-Me-12** (Table 1). All of these new synthetic compounds had GI_50_ values in the 0.5–20 μM range, which were much lower than that of **GA** (>40 μM). The activity order among these compounds was cyano-enone amides **9** > 2-hydroxy- methylene-3-keto compounds **5** > isoxazoles **7** and 2-cyano-3-keto compounds **8**. Since compounds **8a-8d** have decreased activities comparing to that of compounds **9a-9d**, it suggests that a double bond between C-1 and C-2 is required for maintaining high activity in these cyano-enone amides. Compounds **9a**, **9c** and **9d** have activities similar to that of **CDODO-Me-11**. The antiproliferative activity of *N*-(2-cyano-3,11-dioxoolean-1,12-dien-30-yl)-4-piperidyl piperidine (**9b**) is two-fold more than that of **CDODO-Me-11**, indicating that the methyl ester could be replaced to further improve the antiproliferative activities of these compounds. Although **9b** has lower activity than that of **CDODO-Me-12**, which has a 12-oxo-9(11)-en structure in the C-ring, the simple synthesis of **9b** compared to that of **CDODO-Me-12** makes this compound potentially more useful. The antitumor effects of compound **9b** in additional cell lines are worthy of further study.

**Table 1:** The antiproliferative activities of target compounds in HL-60 cells.

Compd.	R	IG_50_(μM)*	Compd.	R	IG_50_(μM)*
**5a**	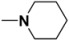	**5.5 ± 0.2**	**8c**	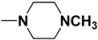	**17.9 ± 1.0**
**5b**	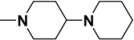	**3.3 ± 0.3**	**8d**	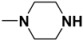	**11.9 ± 0.9**
**5c**	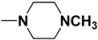	**6.1 ± 0.3**	**9a**	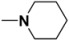	**1.4 ± 0.4**
**7a**	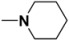	**1.7 ± 0.4**	**9b**	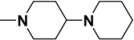	**0.8 ± 0.1**
**7b**	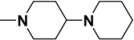	**7.7 ± 0.1**	**9c**	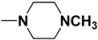	**1.2 ± 0.2**
**7c**	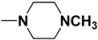	**7.9 ± 0.2**	**9d**	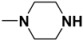	**1.7 ± 0.1**
**7d**	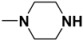	**8.2 ± 0.2**	**GA**		**>40**
**8a**	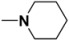	**8.6 ± 0.9**	**CDODO-Me-11**		**1.5 ± 0.1**
**8b**	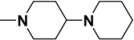	**7.5 ± 0.7**	**CDODO-Me-12**		**0.4 ± 0.1**

* The IG_50_ values were obtained in HL-60 cells after treatments with various concentrations of these compounds for 3 days. The data shown are the mean plus standard deviation (*SD*) of three independent experiments.

## 3. Experimental

### 3.1. General

GA (purity over 98%) was purchased from Shanghai Haokang Chemicals Co. Ltd., China. Other reagents were bought from commercial suppliers in analytic grade and used without further purification, unless noted otherwise. ^1^H-NMR and ^13^C-NMR spectra were recorded on a Bruker ARX-300 instrument with tetramethylsilane as an internal standard. Infra-red (IR) spectra were recorded on a Bruker IR-27G spectrometer. Mass spectra (MS) were determined on a Finnigan MAT/USA spectrometer (LC-MS). HRMS spectra were obtained on a Bruker micrOTOF-Q in an ESI mode. The melting points were determined on an electrically heated X4 digital visual melting point apparatus and are uncorrected. TLC plates (Alugram silica gel G/UV254) were purchased from Macherey-Nagel GmbH & Co. 

*3,11-Dioxoolean-12-en-30-oic acid* (**1**). Jones’ reagent (15 mL) was added to a solution of GA (10.0 g, 21.2 mmol) in THF (35 mL). The solution was stirred at 0 ºC for 1 h and then was poured into H_2_O (100 mL). The precipitate was filtered and dried to afford compound **2** (9.5 g, 95.6% yield) as a white solid. ^1^H- NMR (CDCl_3_) δ (ppm): 5.76 (1H, s, H-12), , 2.97–2.94 (1H, m, H-1), 2.63–2.61 (1H, m, H-1), 2.45 (1H, s, H-9), 1.38, 1.27, 1.23, 1.17, 1.10, 1.07, 0.86 (s, CH_3_×7); LC-MS: 469.4 [M+H]^+^, 591.3 [M+Na]^+^.

*Benzyl 3,11-dioxoolean-12-en-30-oate* (**2**). BnBr (1.29 mL, 10.9 mmol) was added to a solution of **1** (5.0 g, 10.7 mmol) in DMF (50 mL). The mixture was stirred at 100 ºC for 3 h, cooled to room temperature and then poured into water (100 mL). The precipitate was filtered, washed with water to pH 7 and dried to give **2** (4.8 g, 80.9% yield) as a white solid. ^1^H-NMR (CDCl_3_) δ (ppm): 7.40–7.31 (5H, m, -CH_2_Ph), 5.58 (1H, s, H-12), 5.21 (1H, d, *J* = 12.3 Hz, -CH_2_Ph), 5.08 (1H, d, *J* = 12.3 Hz, -CH_2_Ph), 2.97–2.94 (1H, m, H-1), 2.42 (1H, s, H-9), 2.37–2.33 (1H, m, H-1), 1.40, 1.36, 1.27, 1.16, 1.15, 1.06, 0.75 (s, CH_3_×7); ^13^C-NMR (CDCl_3_): δ (ppm) 216.9 (C-3), 199.1 (C-11), 176.7 (C-30), 170. 2 (C-13), 136.0 (C-12), 128.5 (Ph), 128.3 (Ph), 128.2 (Ph), 128.1 (Ph), 101.1 (C-2); LC-MS: 559.4 [M+H]^+^, 581.4 [M+Na]^+^.

*Benzyl 2-hydroxymethylene-3,11-dioxoolean-12-en-30-oate* (**3**). NaH (3.84 g, 160 mmol) was added slowly to a solution of **2** (5.6 g, 10.1 mmol) in ethyl formate (100 mL). The mixture was stirred at r.t. for 10 min and methanol was added until no bubbles were generated. The mixture was diluted with a mixture of CH_2_Cl_2_ and Et_2_O (1:2) and washed three times with 5% aqueous HCl solution. The organic layer was evaporated in vacuum and purified on a silica gel column with cyclohexane-acetone (v/v) = 20:1 to give **3 **(4.7 g, 80.0% yield) as a white solid. ^1^H-NMR (CDCl_3_) δ (ppm): 14.89 (s, 1H, OH), 8.61 (s, 1H, =CH-OH), 7.40-7.34 (m, 5H, -CH_2_Ph), 5.60 (s, 1H, H-12), 5.21 (d, 1H, *J* = 12.3 Hz, -CH_2_Ph), 5.07 (d, 1H, *J* = 12.3 Hz, -CH_2_Ph), 3.45 (d, 1H, *J* = 14.7 Hz, H-1), 2.44 (s, 1H, H-9), 1.35, 1.19, 1.16, 1.15, 1.13, 1.12, 0.75 (s, CH_3_×7); ^13^C-NMR (CDCl_3_): δ (ppm) 199.7 (C-11), 189.8 (C-3), 189.1 (C=CH-OH), 176.4 (C-30), 170.1 (C-13), 128.9 (Ph), 128.8 (Ph), 128.6 (Ph), 128.5 (-Ph), 128.3 (C-12), 106.1 (C-2), 66.4 (-CH_2_Ph), 59.8 (C-9); LC-MS: 585.3 [M-H]^-^.

*2-Hydroxymethylene-3,11-dioxoolean-12-en-30-oic acid* (**4**). 10% Pd/C (0.14 g) was added to a solution of **3** (0.76 g, 1.3 mmol) in THF (20 mL). The mixture was stirred at r.t. for 8 hr and filtered to remove Pd/C. The filtrate was concentrated in vacuum and the residue was purified on a silica gel column with petroleum ether : acetone (v/v) = 20:1 to give **4 **(0.49 g, 76.3% yield) as a pink solid. ^1^H-NMR (CDCl_3_) δ (ppm): 14.89 (s, 1H, =CH-OH), 8.64 (s, 1H, =CH-OH), 5.79 (s, 1H, H-12), 3.47 (d, 1H, *J* = 15.0 Hz, H-1), 2.46 (s, 1H, H-9), 1.39, 1.25, 1.21, 1.19, 1.15, 1.14, 0.88 (s, CH_3_×7); ^13^C-NMR (CDCl_3_): δ (ppm) 200.0 (C-11), 189.8 (C-3), 189.2 (C=CH-OH), 182.7 (C-30), 170.3 (C-13), 128.8 (C-12), 106.1 (C-2), 59.9 (C-9); LC-MS: 495.4 [M-H]^-^.

### 3.2. General procedure for the preparation of 2-hydroxymethylene-30-amides **5a-5c**

To a solution of **4** (2.34 g, 4 mmol) in chloroform (10 mL), oxalyl chloride (2 mL) was added. The mixture was stirred at r.t. for 1 hr and excess oxalyl chloride was removed by co-evaporation with hexane (three times). The obtained solid was dissolved in chloroform (30 mL) and an appropriate nitrogen heterocycle (6 mmol) was added. The mixture was stirred at r.t. for 5 min and washed three times with 5% aqueous HCl solution. The organic layer was dried over anhydrous MgSO_4_. After MgSO_4_ was removed by filtration, the filtrate was concentrated in vacuum and the residue was purified on a silica gel column with chloroform-methanol (v/v) = 50:1 to give a white solid. The *Rf* values were determined using TLC plates with chloroform-methanol (v/v) = 10:1.

*N-(2-Hydroxymethylene-3,11-dioxoolean-12-en-30-yl)-piperidine* (**5a**). Crystallization of the white solid from hexane and acetone (10:1) afforded white needles (1.6 g, 2.9 mmol). Yield: 72%; *R_f _*= 0.4; m.p. 178–181 ºC; IR (KBr): 3425, 2927, 2867, 1612, 1446, 1417, 1382, 1362 cm^-1^; ^1^H-NMR (CDCl_3_): δ (ppm) 14.89 (d, 1H, *J* = 3.0 Hz, =CH-OH), 8.64 (d, 1H, *J* = 3.0 Hz, =CH-OH), 5.80 (s, 1H, H-12), 3.65-3.53 (m, 4H), 3.48 (d, 1H, *J* = 14.1 Hz, H-1), 2.45 (s, 1H, H-9), 1.38, 1.23, 1.21, 1.18, 1.15, 1.14, 0.85 (s, CH_3_×7); ^13^C-NMR (CDCl_3_): δ (ppm) 200.4 (C-11), 189.8 (C-3), 189.2 (CH=CH-OH), 174.0 (C-30), 171.4 (C-13), 129.8 (C-12), 106.1 (C-2), 59.9 (C-9); LC-MS: 562.6 [M-H]^-^; HRMS: *m/z*, calcd. for C_36_H_52_NO_4 _(M-H) 562.3890. Found: 562.3882. 

*N-(2-Hydroxymethylene-3,11-dioxoolean-12-en-30-yl)-4-piperidyl piperidine* (**5b**). Crystallization of the white solid from hexane and acetone (10:1) afforded white needles (1.8 g, 2.8 mmol). Yield: 70%; *R_f_ =* 0.3; m.p. 210–212 ºC; IR (KBr): 3426.1, 2947.7, 2650.1, 2527.8, 1633.5, 1456.8 cm^-1^; ^1^H-NMR (CDCl_3_): δ (ppm) 14.89 (d, 1H, *J* = 3.0 Hz, =CH-OH), 8.63 (d, 1H, *J* = 3.0 Hz, =CH-OH), 5.73 (s, 1H), 4.54 (br, 2H), 3.45 (d, 1H, *J* = 14.7 Hz, H-1), 3.06-2.70 (m, 10H), 2.44 (s, 1H, H-9), 1.38, 1.23, 1.21, 1.17, 1.15, 1.13, 0.84 (s, CH_3_×7); LC-MS: 665.4 [M+NH_4_]^+^; HRMS: *m/z*, calcd. for C_41_H_63_N_2_O_4 _(M+H) 647.4782. Found: 647.4783. 

*N-(2-Hydroxymethylene-3,11-dioxoolean-12-en-30-yl)-4-methyl piperazine* (**5c)**. Crystallization of the white solid from hexane and acetone (5:1) afforded white needles (1.6 g, 2.7 mmol). Yield: 68%; *R_f _=* 0.2; m.p. 155–157 ºC; IR (KBr): 3439, 2951, 2587, 1636, 1462, 1406, 1384 cm^-1^; ^1^H-NMR (CDCl_3_): δ (ppm) 14.90 (d, 1H, *J* = 3.0 Hz, =CH-OH), 8.64 (d, 1H, *J* = 3.0 Hz, =CH-OH), 5.80 (s, 1H), 3.67 (br, 4H), 3.49 (d, 1H, *J* = 14.7 Hz, H-1), 2.50–2.38 (m, 5H), 2.33 (s, 3H, N-CH_3_), 1.38, 1.23, 1.21, 1.18, 1.15, 1.14, 0.85 (s, CH_3_×7); LC-MS: 577.4 [M-H]^-^; HRMS: *m/z*, calcd. for C_36_H_54_N_2_O_4 _(M-H) 577.3999. Found: 577. 3990.

*Isoxazolo[4, 5-b]olean-11-oxo 12-en-30-oic acid* (**6**). A mixture of compound **4** (0.89 g, 0.9 mmol), NH_2_OH**^.^**HCl (0.36 g, 5.1 mol), and anhydrous sodium acetate (0.04 g, 0.49 mmol) were refluxed for 1.5 h in acetic acid (20 mL), cooled, and poured into ice-water. The precipitate was filtered, dried, and purified on a silica gel column with petroleum ether-acetone (v/v) = 20:1 to give **6 **(0.74 g, 85.0% yield) as a white solid. ^1^H-NMR (CDCl_3_) δ (ppm): 12.07 (brs, 1H, -COOH), 8.03 (s, 1H, -CH=N-), 5.77 (s, 1H, H-12), 3.64 (d, 1H, *J* = 15.3 Hz, H-1), 2.56 (s, 1H, H-9), 1.41, 1.27, 1.25, 1.21, 1.18, 1.09, 0.86 (s, CH_3_×7); LC-MS: 492.4 [M-H]^-^.

### 3.3. General procedure for the preparation of isoxazolo[4,5-b]olean-11-oxo-12-en-30-amides **7a-7d**

Oxalyl chloride (2 mL) was added to a solution of 6 (0.98 g, 2 mmol) in chloroform (10 mL). The mixture was stirred at r.t. for 1 hr and excess oxalyl chloride was removed by evaporation with hexane (three times). The obtained solid was dissolved in 30 mL chloroform and a nitrogen heterocycle (6 mmol) was added. The mixture was stirred at r.t. for 5 min and concentrated under vacuum. The residue was purified on a silica gel column with chloroform-methanol (v/v) = 50:1 to give a white solid. The Rf values were determined by TLC plates with chloroform-methanol (v/v) = 10:1.

*N-(Isoxazolo[4,5-b]olean-11-oxo-12-en-30-yl)-piperidine* (**7a**). Crystallization of the white solid from hexane afforded an amorphous solid (0.67 g, 60% yield). m.p. 148–151 ºC; *R_f_ =* 0.4; IR (KBr): 3429, 2929, 2853, 1628, 1462, 1411, 1383 cm^-1^; ^1^H-NMR (CDCl_3_): δ (ppm) 7.99 (s, 1H, -CH=N-), 5.81 (s, 1H, H-12), 3.65 (d, 1H, *J* = 15.7 Hz, H-1), 3.59 (br, 4H), 2.54 (s, 1H, H-9), 2.05 (m, 4H), 1.42, 1.38, 1.32, 1.24, 1.22, 1.18, 0.88 (s, CH_3_×7); ^13^C-NMR (CDCl_3_): δ (ppm) 199.3 (C-11), 173.6 (C-30), 172.2 (C-13), 170.4 (C-3), 150.3 (C-C=N), 128.6 (C-12), 109.1 (C-2); LC-MS: 559.4 [M-H]^-^; HRMS: *m/z*, calcd. for C_36_H_5__1_N_2_O_3 _(M-H) 559.3894. Found: 559.3903. 

*N-(Isoxazolo[4,5-b]olean-11-oxo-12-en-30-yl)-4-piperidyl piperidine* (**7b)**. Crystallization of the white solid from hexane afforded an amorphous solid (0.86 g, 67% yield). m.p. 158–162 ºC; *R_f_ =* 0.2; IR (KBr): 3439, 2932, 2853, 1724, 1630, 1456, 1413, 1383 cm^-1^; ^1^H-NMR (CDCl_3_): δ (ppm) 7.99 (s, 1H, -CH=N-), 5.79 (s, 1H, H-12), 4.51 (br, 2H), 3.65 (d, 1H, *J* = 15.7 Hz, H-1), 2.56 (s, 1H, H-9), 1.38, 1.32, 1.24, 1.22, 1.18, 1.10, 0.83 (s, CH_3_×6); ^13^C-NMR (CDCl_3_): δ (ppm) 200.7 (C-11), 173.6 (C-30),172.2 (C-13), 170.3 (C-3), 150.4 (C-C=N), 128.6 (C-12), 109.1 (C-2); LC-MS: 644.4 [M+H]^+^; HRMS: *m/z*, calcd. for C_41_H_6__2_N_3_O_3 _(M+H) 644.4785. Found: 643.4717. 

*N-(Isoxazolo[4,5-b]olean*-*11-oxo -12-en-30-yl)-4-methyl piperazine* (**7c**). Crystallization of the white solid from hexane afforded an amorphous solid (0.58 g, 51% yield). m.p. 157–159 ºC; *R_f_ =* 0.2; IR (KBr): 3436, 2939, 2790, 1722, 1633, 1460, 1410.8, 1383.5 cm^-1^; ^1^H-NMR (CDCl_3_): δ (ppm) 7.99 (1H, s, -CH=N-), 5.81(s, 1H, H-12), 3.74 (br, 4H), 3.65 (d, 1H, *J* = 15.7 Hz, H-1), 2.53 (s, 3H, N-CH_3_), 2.41 (br, 4H), 1.38, 1.32, 1.24, 1.22, 1.18, 1.10, 0.85 (s, CH_3_×7); LC-MS: 576.5 [M+H]^+^, 598.5 [M+Na]^+^; HRMS: *m/z*, calcd. for C_36_H_5__4_N_3_O_3 _(M+H) 576.4159. Found: 576.4153. 

*N-(Isoxazolo[4,5-b]olean-11-oxo-12-en-30-yl)-piperazine* (**7d**). Crystallization of the white solid from hexane afforded an amorphous solid (0.26 g, 50% yield). m.p. 216–218 ºC; *R_f_ =* 0.1; IR (KBr): 3427, 2968, 2866, 2467, 1725, 1637**, **1459, 1409 cm^-1^; ^1^H-NMR (CDCl_3_): δ (ppm) 7.99 (1H, s, -CH=N-), 5.80 (s, 1H, H-12), 3.68 (m, 4H), 3.65 (d, 1H, *J* = 15.3 Hz, H-1), 2.91 (m, 4H), 2.54 (s, 1H, H-9), 1.38, 1.32, 1.24, 1.22, 1.18, 1.10, 0.85 (s, CH_3_×7); LC-MS: 562.3 [M+H]^+^; HRMS: *m/z*, calcd. for C_35_H_5__2_N_3_O_3 _(M+H) 562.4003. Found: 562.3999. 

### 3.4. General procedure for the preparation of 2-cyano-3,11-dioxoolean-12-en-30-amides **8a-8d**

NaOCH_3_ (7.25 g, 134 mmol) was added to a solution of isoxazolo[4,5-*b*]-olean-11-oxo-12-en- 30-amide (3.9 mmol) in MeOH (60 mL) and Et_2_O (125 mL). The mixture was stirred at r.t. for 8 hr and was extracted with a mixture of CH_2_Cl_2_ and Et_2_O (1:2). The extract was washed three times with 5% aqueous HCl solution and the acidic washings were re-extracted with a mixture of CH_2_Cl_2_ and Et_2_O (1:2). The combined organic layers were evaporated in vacuum and the precipitate was purified on a silica gel column with chloroform-methanol (v/v) = 50:1 to give a white solid. The *Rf* values were determined by TLC plates with chloroform-methanol (v/v) = 10:1.

*N-(2-Cyano-3,11-dioxoolean-12-en-30-yl)-piperridine* (**8a**). Crystallization of the white solid from hexane and EtOAc (10:1) afforded an amorphous solid (1.81 g, 83% yield). m.p. 163–165 ºC; *R_f_ =* 0.4; IR (KBr): 3434, 2931, 2855, 2359, 2204, 1722, 1629 cm^-1^; ^1^H-NMR (CDCl_3_): δ (ppm) 5.77 (s, 1H, H-12), 3.89 (m, 1H, H-2), 3.57 (br, 4H), 3.32 (m, 1H, H-1), 2.37 (s, 1H, H-9), 2.08 (m, 6H), 1.39, 1.35, 1.34, 1.22, 1.21, 1.10, 0.83 (s, CH_3_×7); ^13^C-NMR (CDCl_3_): δ (ppm) 207.4 (C-3), 197.8(C-11), 173.5(C-30), 171.3(C-13), 128.3 (C-12), 117.1(-CN), 79.6 (C-2), 61.3 (C-9); LC-MS: 559.4 [M-H]^-^; HRMS: *m/z*, calcd. for C_36_H_53_N_2_O_3 _(M-H) 559.3894. Found: 559.3898.

*N-(2-Cyano-3,11-dioxoolean-12-en-30-yl)-4-piperidyl piperidine* (**8b**). Crystallization of the white solid from hexane and EtOAc (10:1) afforded an amorphous solid (1.91 g, 76% yield). m.p. 189–191 ºC; *R_f_ =* 0.3; IR (KBr): 3423, 2948, 2647, 2528, 2202, 1627, 1457, 1417 cm^-1^; ^1^H-NMR (CDCl_3_): δ (ppm) 5.74 (s, 1H, H-12), 4.53 (br, 2H), 3.96 (m, 1H, H-2), 3.55 (m, 1H, H-1), 2.88-2.76 (br, 8H), 2.44-2.38 (m,4H), 2.36 (s, 1H, H-9),1.37, 1.35, 1.22, 1.18, 1.16, 1.11, 0.82 (s, CH_3_×7); ^13^C-NMR (CDCl_3_): δ (ppm) 205.2 (C-3), 198.6 (C-11), 173.8 (C-30), 170.8 (C-13), 128.1 (C-12), 117.1 (-CN), 79.5 (C-2), 63.1 (C-9); LC-MS: 644.7 [M+H]^+^; HRMS: *m/z*, calcd. for C_41_H_62_N_3_O_3 _(M+H) 644.4785. Found: 644.4789. 

*N-(2-Cyano-3,11-dioxoolean-12-en-30-yl)-4-methyl piperazine*** (8c**). Crystallization of the white solid from hexane and EtOAc (10:1) afforded an amorphous solid (1.50 g, 67% yield). m.p. 197–199 ºC; *Rf =* 0.2; IR (KBr): 3425, 2947, 2792, 2202, 1719, 1633, 1460, 1410, 1383 cm^-1^; ^1^H-NMR (CDCl_3_): δ (ppm) 5.78 (s, 1H, H-12), 3.97 (m, 1H, H-2), 3.80 (br, 4H), 3.59 (m, 1H, H-1), 2.55 (s, 3H, N-CH_3_), 2.54 (s, 1H, H-9), 1.43, 1.38, 1.35, 1.18, 1.17, 1.15, 0.83 (s, CH_3_×7); ^13^C-NMR (CDCl_3_): δ (ppm) 205.2 (C-3), 198.6 (C-11), 173.9 (C-30), 170.7 (C-13), 128.2 (C-12), 117.1(-CN), 60.6 (C-9); LC-MS: 576.4 [M+H]^+^; HRMS: *m/z*, calcd. for C_36_H_54_N_3_O_3 _(M+H) 576.4159. Found: 576.4160. 

*N-(2-cyano-3,11-dioxoolean-12-en-30-yl)-piperazine* (**8d**). Crystallization of the white solid from hexane and EtOAc (10:1) afforded an amorphous solid (0.88 g, 60% yield). m.p. 260–263 ºC; *R_f _=* 0.1; IR (KBr): 3423, 2950, 2466, 2203, 1718, 1633, 1459 cm^-1^; ^1^H-NMR (CDCl_3_): δ (ppm) 5.77 (s, 1H, H-12), 3.97 (m, 1H, H-2), 3.71 (br, 4H), 3.62 (m, 1H, H-1), 2.95 (br, 4H), 2.41 (s, 1H, H-9), 1.43, 1.38, 1.35, 1.23, 1.18, 1.09, 0.83 (s, CH_3_×7); LC-MS: 562.6 [M+H]^+^; HRMS: *m/z*, calcd. for C_35_H_52_N_3_O_3 _(M+H) 562.4003. Found: 562.4009. 

### 3.5. General procedure for the preparation of 2-cyano-3,11-dioxoolean-1,12-dien-30-amide derivatives **9a-9d**

A mixture of 2-cyano-3,11-dioxoolean-12-en-30-amide derivative (3 mmol) and DDQ (98%) (0.77 g, 3.32 mmol) in dry toluene (80 mL) was heated under 80 ºC for 30 min. After insoluble material was removed by filtration, the filtrate was evaporated under vacuum to give a brown solid. The solid was purified on a silica gel column with chloroform-methanol (v/v) = 50:1. The *R_f_* values were determined by TLC plates with chloroform-methanol (v/v) = 10:1.

*N-(2-Cyano-3,11-dioxoolean-1,12-dien-30-yl)-piperidine* (**9a**). Crystallization of the white solid from hexane and EtOAc (10:1) afforded an amorphous solid (0.94 g, 56% yield). m.p. 187–190 ºC; *R_f_ =* 0.4; IR (KBr): 3428, 2933, 2856, 2233, 1687, 1626, 1462 1412 cm^-1^; ^1^H-NMR (CDCl_3_): δ (ppm) 8.41 (s, 1H, H-1), 5.74 (s, 1H, H-12), 3.57 (br, 4H), 2.61 (s, 1H, H-9), 1.51, 1.37, 1.29, 1.21, 1.20, 1.15, 0.85 (s, CH_3_×7); ^13^C-NMR (CDCl_3_): δ (ppm) 197.6 (C-3), 197.5 (C-11), 176.1 (C-30), 172.5 (C-13), 168.5 (C-1), 123.4 (C-12), 114.8 (-CN), 113.3 (C-2), 53.4 (C9); LC-MS: 557.4 [M-H]^-^, 593.5 [M+Cl]^-^; HRMS: *m/z*, calcd. for C_36_H_50_N_2_O_3 _(M-H) 557.3737. Found: 5578.3733. 

*N-(2-Cyano-3,11-dioxoolean-1,12-dien-30-yl)-4-piperidyl piperidine* (**9b**). Crystallization of the white solid from hexane and EtOAc (10:1) afforded an amorphous solid (0.98 g, 51% yield). m.p. 218–220 ºC; *R_f_ =* 0.3; IR (KBr): 3431, 2949, 2524, 2233, 1684, 1627, 1416 cm^-1^; ^1^H-NMR (CDCl_3_): δ (ppm) 8.40 (s, 1H, H-1), 5.73 (s, 1H, H-12), 4.58 (br, 2H), 3.14 (m, 1H), 2.85-2.78 (m, 4H), 2.61 (s, 1H, H-9), 2.27 (br, 4H), 1.51, 1.37, 1.31, 1.20, 1.19, 1.15, 0.83 (s, CH_3_×7); ^13^C-NMR (CDCl_3_): δ (ppm) 197.6 (C-3), 197.5 (C-11), 176.4 (C-30), 172.4 (C-13), 168.2 (C-1), 123.5 (C-12), 114.9 (-CN), 113.3(C-2); LC-MS: 642.6 [M+H]^+^; HRMS: *m/z*, calcd. for C_41_H_60_N_3_O_3 _(M+H^+^) 642.4629. Found: 642.4626.

*N-(2-Cyano-3,11-dioxoolean-1,12-dien-30-yl)-4-methyl piperazine* (**9c**). Crystallization of the white solid from hexane and EtOAc (10:1) afforded an amorphous solid (0.74 g, 43% yield). m.p. 185–187 ºC; *R_f_ =* 0.3; IR (KBr): 3438, 2950, 2680, 1719, 1685, 1633, 1461, 1408, 1384 cm^-1^; ^1^H-NMR (CDCl_3_): δ (ppm) 8.40 (s, 1H, H-1), 5.73 (s, 1H, H-12), 3.81 (br, 4H), 2.64 (br, 4H), 2.47 (s, 3H, -NCH_3_), 1.51, 1.38, 1.30, 1.21, 1.20, 1.15, 0.74 (s, CH_3_×7); LC-MS: 574.5[M+H]^+^; HRMS: *m/z*, calcd. for C_36_H_52_N_3_O_3 _(M+H) 574.4003. Found: 574.3997. 

*N-(2-Cyano-3,11-dioxoolean-1,12-dien-30-yl)-piperazine* (**9d**). Crystallization of the white solid from hexane and EtOAc (10:1) afforded an amorphous solid (0.59 g, 40% yield). m.p. 201–203 ºC; *R_f_ =* 0.1; IR (KBr): 3426, 2951, 2468, 2233, 1721, 1686, 1638, 1459, 1384 cm^-1^; ^1^H-NMR (CDCl_3_): δ (ppm) 8.39 (s, 1H, H-1), 5.72 (s, 1H, H-12), 3.65 (br, 4H), 2.91 (br, 4H), 2.61 (s, 1H, H-9), 1.51, 1.49, 1.37, 1.30, 1.21, 1.20, 1.15, 0.74 (s, CH_3_×7); ^13^C-NMR (CDCl_3_): δ (ppm) 197.3 (C-3), 197.2 (C-11), 176.1 (C-30), 172.1 (C-13), 167.9 (C-1), 123.2 (C-12), 114.6 (-CN), 113.1 (C-2); LC-MS: 560.5 [M+H]^+^; HRMS: *m/z*, calcd. for C_35_H_50_N_3_O_3 _(M+H) 560.3846. Found: 560.3853. 

### 3.6. Cell culture

HL-60 cells were cultured in RPMI 1640. The media were supplemented with 100 units/mL penicillin, 100 μg/mL streptomycin, 1 mmol/L L-glutamine, and 10% (v/v) heat-inactivated fetal bovine serum.

### 3.7. Cell growth inhibition assay

All compounds were dissolved in DMSO. A stock solution of 20 mmol/L of each compound was prepared in DMSO and stored in aliquots at -20 ºC. A working solution was diluted with ethanol and fresh medium before assaying. The final concentration of ethanol in the medium was less than 1% and the final concentration of DMSO was less than 0.1%. Cells were seeded at a density of 4 × 10^4 ^cells/mL in 24 well plates with various concentrations of the tested compounds and incubated for 3 days. Total cell number in each group was determined using a hemocytometer. The cell growth inhibitory ability was expressed as the ratio of the cell number in the groups treated with the compounds to that of cells treated with DMSO and/or ethanol. The concentration (GI_50_) which inhibited half of cell growth was calculated.

## 4. Conclusions

Fifteen novel glycyrrhetinic acid derivatives containing a nitrogen heterocycle at C-30 and with different substituents on the A-ring were designed and synthesized. The antiproliferative effects of these compounds were determined in HL-60 cells. The results reveal that: 1) introduction of the cyano-enone moiety into the A-ring of **GA** significantly improves the antiproliferative activities in leukemia cells and 2) *N*-(2-cyano-3,11-dioxoolean-1,12-dien-30-yl)-4-piperidyl piperidine (**9b**) is the most active compound among these novel **GA** derivatives with a 11-oxo-12-en structure.
